# A comprehensive study on the structure, microstructure and electrical properties of a Dy-doped LaBiO_3_ ceramic for thermistor application

**DOI:** 10.1039/d5ra06969f

**Published:** 2026-01-26

**Authors:** Chandan Kumar Lenka, S. K. Parida

**Affiliations:** a Department of Physics, ITER, Siksha ‘O’ Anusandhan Deemed to be University Bhubaneswar-751030 India santoshparida@soa.ac.in

## Abstract

In this work, the synthesis of dysprosium (Dy)-substituted lanthanum bismuth oxide (LaBi_0.95_Dy_0.05_O_3_, LBDO) *via* solid-state reaction and its structural, microstructural, dielectric, and conductivity characterizations are systematically investigated. MADU Rietveld refinement of the X-ray diffraction data confirms that the compound exhibits an orthorhombic crystal structure at room temperature, with an average crystallite size of 49.1 nm, micro lattice strain of 0.000876, and dislocation density of 4.14 × 10^14^ m^−2^. Scanning electron microscopy (SEM) analysis reveals a uniform grain distribution across the sample surface, which may contribute to improved electrical conductivity. Furthermore, energy-dispersive X-ray spectroscopy (EDX) verifies the presence of all constituent elements (La, Bi, Dy, and O) in both weight and atomic percentages within the prepared material. The investigation of dielectric properties as a function of temperature and frequency indicates the presence of Maxwell–Wagner type dielectric dispersion. Impedance analysis confirms the negative temperature coefficient of resistance (NTCR) behavior, while modulus spectroscopy reveals a non-Debye type relaxation process. The ac conductivity study suggests a thermally activated hopping mechanism in the material. Moreover, both Nyquist and Cole–Cole plot analyses validate its semiconducting nature, making it a promising candidate for energy storage device applications. Additionally, the resistance–temperature relationship confirms an NTC thermistor characteristic, highlighting its potential use in temperature sensor devices.

## Abstract 2

In this work, the synthesis of dysprosium (Dy)-substituted lanthanum bismuth oxide (LaBi_0.95_Dy_0.05_O_3_, LBDO) *via* solid-state reaction and its structural, microstructural, dielectric, and conductivity characterizations are systematically investigated.

## Graphical abstract

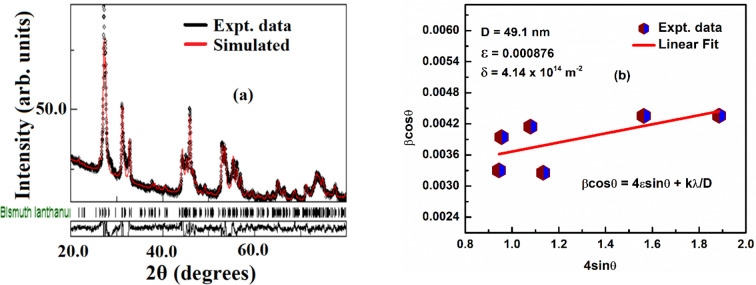

## 1. Introduction

Single perovskite materials are widely studied due to their remarkable structural flexibility, tunable physical properties, and broad applicability in electronics, optoelectronics, and energy storage devices. Their simple ABO_3_ structure allows for systematic substitution at A- or B-sites, enabling controlled modification of dielectric, ferroelectric, and electrical behavior. Studying single-phase perovskites provides clearer insights into intrinsic mechanisms without the complications arising from multiphase systems. These materials exhibit excellent dielectric properties, high thermal stability, and low dielectric loss, making them suitable for capacitors, sensors, and actuators.^1–5^ Research also emphasizes their role in replacing lead-based materials with eco-friendly alternatives. Overall, single perovskite systems serve as ideal models for understanding structure–property relationships essential for designing next-generation functional materials.

Doped single perovskite ceramic materials are increasingly significant due to their tunable physical and functional properties, making them suitable for a wide range of electronic and energy storage applications. Doping allows modification of the crystal structure, electrical conductivity, dielectric behavior, and thermal stability of the base perovskite. By introducing aliovalent or isovalent ions into the A- or B-sites of the perovskite lattice (ABO_3_), properties like dielectric constant, loss tangent, impedance response, and band gap can be precisely engineered. For instance, rare-earth doping (*e.g.*, Gd, Er, Nd, Sm) in LaBiO_3_ ceramics improves their dielectric performance and conduction mechanisms, making them promising candidates for capacitors, sensors, and optoelectronic devices.^6–13^ Wang *et al.* investigated the photocatalytic properties of lanthanum bismuth oxide through CO_2_ reduction. Their study found that La–Bi–O compounds (La_0.225_Bi_0.775_O_1.5_, La_0.6_Bi_0.4_O_1.5_, and La_1.08_Bi_0.92_O_3.03_) exhibit promising semiconductor behavior, demonstrating excellent potential, stability, and efficiency for photocatalytic applications.^14^ Yun *et al.* conducted an in-depth investigation into the luminescence properties of dysprosium (Dy^3+^)-doped double perovskite tellurite compounds and predicted their use in white-light emitting devices and other luminescent applications.^15^

According to existing research, rare-earth-doped perovskites show a broader range of electrical and optical properties compared to their parent perovskites, suggesting enhanced application potential. Although extensive research has been conducted on Dy-doped perovskite materials,^16–22^ detailed investigations on lanthanum bismuth oxide perovskites remain limited, particularly at low concentrations of Dy. Therefore, in this study, 5% of Dy-modified lanthanum bismuth oxide was synthesized *via* the solid-state reaction method to examine its structural and electrical characteristics and to assess its suitability for various scientific and industrial applications. The results demonstrate promising electrical behavior, indicating that this material could serve as a potential candidate for NTC thermistor-based temperature sensing devices.

## 2. Experimental details

### 2.1 Raw materials required

High-purity starting materials; Bi_2_O_3_ (99.9%), Dy_2_O_3_ (99.0%), and La_2_O_3_ (99.0%) were selected for synthesizing of the LaBi_0.95_Dy_0.05_O_3_ ceramic. These analytical reagents (AR) grade chemicals with high purity levels were procured from Loba Chemie Pvt. Ltd. The purchased metal oxide powders were weighed in a stoichiometric ratio by using a digital balance [Mettler Type; New classic MF Model; ML 204/A01] with a standard error bar of 0.0001. The required chemical equation for the synthesis of the final product is given as: 0.475Bi_2_O_3_ + 0.025Dy_2_O_3_ + 0.5La_2_O_3_ → LaBi_0.95_Dy_0.05_O_3_.

### 2.2 Synthesis and sintering

The weighed metal oxide powders were thoroughly mixed using an agate mortar and pestle. Initially, dry grinding was carried out for 2 hours, followed by wet grinding with methanol for another 2 hours to ensure better homogeneity of the mixture. Grinding was continued until uniform dispersion and nanoscale particle size were achieved. The resulting nano powders were then kept in a high-temperature-resistant crucible and subjected to calcination at 1050 °C for 5–6 hours in a programmable high-temperature furnace. The formation of the desired phase was confirmed using X-ray diffraction (XRD) analysis. The calcined powders were then pressed into cylindrical pellets with diameters of 12 mm and a thickness of 2 mm under a uniaxial hydraulic pressure of 4 MPa. To enhance homogeneity, the pellets were sintered at 1100 °C for 5 hours to achieve higher densification and eliminate residual impurities (carbon and oxygen), if any.

### 2.3 Characterization

The structural properties of the synthesized sample were analyzed using X-ray diffraction (XRD) with a RIGAKU Japan ULTIMA IV system, employing CuKα radiation (*λ* = 1.5405 Å) and a step size = 0.02° used to perform structural investigation at room temperature over a broad range of Bragg's angles (20° ≤ *θ* ≤ 80°). Surface morphology and elemental composition were examined through SEM-EDX analysis using a ZEISS EVO-18 instrument, operated at an accelerating voltage of 2000 kV and a working distance of 9270 µm. Before electrical measurements, both faces of the pellet were coated with conductive silver paint (SPI Supplies Silver Paste) to form uniform, low-resistance electrodes that ensure good electrical contact and homogeneous electric field distribution, thereby minimizing contact resistance and enabling accurate measurement of the intrinsic electrical properties of the sample. The dielectric properties were examined using an impedance analyzer (Model: N4L PSM 1735) over the frequency range of 1 kHz to 1 MHz and the temperature range of 25 °C to 500 °C. The subsequent section presents a detailed discussion of the structural, dielectric, and electrical characteristics of the synthesized perovskite material.

## 3. Results and discussion

### 3.1 Sample formation and XRD analysis

**Fig. 1:**
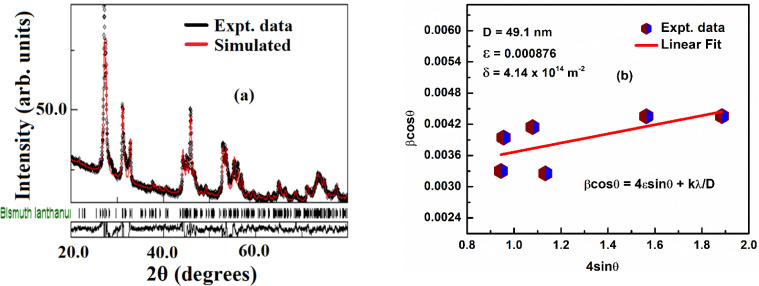
(a) Rietveld refinement and (b) Williamson–Hall plot to calculate the average crystallite size of the LBDO ceramic.

The tolerance factor is a key parameter used to verify the structural stability of the synthesized sample. A typical single perovskite structure is denoted as ABO_3_, where the A-site is generally occupied by trivalent ions and the B-site by trivalent ions. The tolerance factor of LaBi_0.95_Dy_0.05_O_3_ was evaluated using the relation: 
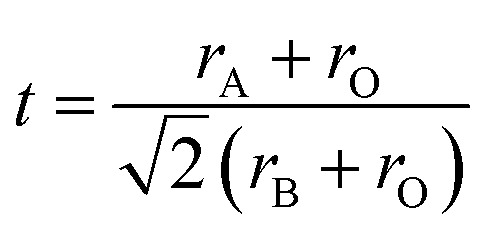
, where *r*_A_ and *r*_B_ represent the ionic radii of the A-site and B-site cations, respectively, and *r*_O_ corresponds to the ionic radius of oxygen.^23^ Tolerance factor of the LaBi_0.95_Dy_0.05_O_3_ ceramic was calculated using proper values of Shannon's ionic radii.^24^ The obtained tolerance factor of 0.82 suggests the formation of a stable orthorhombic structure.^25^ The XRD pattern of the LBDO ceramic is presented in Fig. 1(a), while Fig. 1(b) shows a Williamson–Hall plot to calculate average crystallite size.

In this present study, the structural Rietveld refinement was done using MADU software. In structural refinement, the best possible structure is selected to be orthorhombic crystal symmetry, and lattice constants extracted from refinement for the orthorhombic perovskite phase are: *a* = 12.031567 Å, *b* = 16.368689 Å, *c* = 4.0890946 Å, and *α* = *β* = *γ* = 90°. The reliability factors extracted from refinement are; *R*_wp_ (%) = 13.996441, *R*_exp_ (%) = 4.6324773, *R*_b_ (%) = 10.705353, and *σ* = 3.0213728. The value of the average crystallite size, lattice strain and dislocation density of the prepared sample were calculated using Williamson–Hall (W–H) formula; *β* cos *θ* = 4*ε* sin *θ* + *kλ*/*D*, where *k* = 0.89, *λ* = 0.154 nm, *ε* = lattice strain, *D* = average crystallite size, *β* = full width at half maximum, and *θ* = Bragg's angle.^26^ In the present study, the value of the average crystallite size is found to be 49.1 nm [error bar: 6.38331 × 10^−4^], whereas the value of lattice strain is found to be 0.000876 [error bar: 4.8834 × 10^−4^] in the studied sample. Based on a relation, dislocation density (*δ*) = 1/*D*^2^ can be calculated and found to be 4.14 × 10^14^ m^−2^.

### 3.2 Microstructure study

**Fig. 2:**
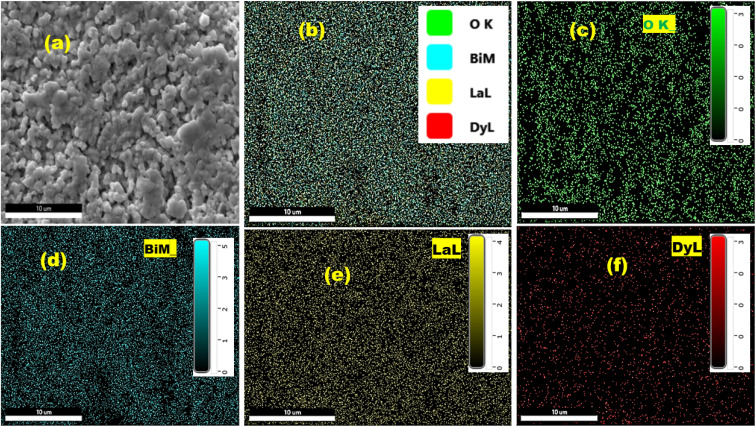
(a–f) Presents the selected area color mapping of the prepared compound, confirming the presence of all constituent elements (La, Bi, Dy, and O).

Fig. 2(a–f) presents the selected area color mapping images with appropriate scaling for the synthesized sample. The mapping indicates a high level of purity, as all elements La, Bi, Dy, and O are distinctly represented by separate colors. The uniform color distribution across the sample suggests an even dispersion of the constituent ions, which likely contributes to the improved physical properties observed in the material.

**Fig. 3:**
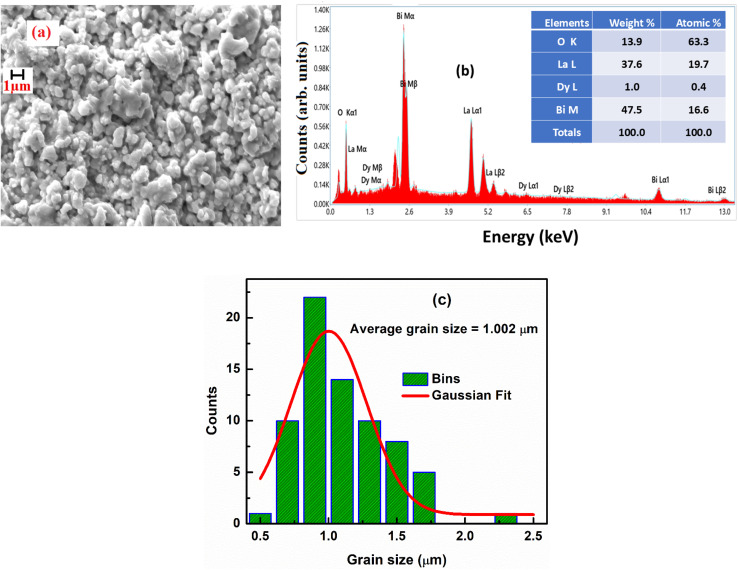
(a) SEM micrograph, (b) EDX spectrum, and (c) grain size calculation using Gaussian fit of the LBDO ceramics.

Fig. 3(a) displays the SEM image of the LaBi_0.95_Dy_0.05_O_3_ ceramic at a resolution of 1 µm. The image reveals a uniform grain distribution across the surface, with clearly defined grain boundaries. ImageJ software was utilized to determine the average grain size. The corresponding EDX spectrum validates the presence of the expected elements within a randomly selected region of the sample. Fig. 3(b) shows distinct peaks representing each element, and the inset provides the weight percentage (wt%), atomic percentage (at%), and net intensity (Net. Int.) for La, Bi, Dy, and O. Fig. 3(c) shows the distribution of different size of grains over the surface of sample in the form of histogram. The average grain size in the sample is found to be 1.002 µm. For uniform and dense grain distribution, the agglomeration rate, *i.e.*, ratio of average grain size to average crystallite, is calculated, which is found to be 35 and supports better conductivity.^27^ This microstructural and compositional analysis confirms the high purity of the synthesized compound, with a complete elemental composition matching the intended stoichiometry.

### 3.3 Dielectric study

**Fig. 4:**
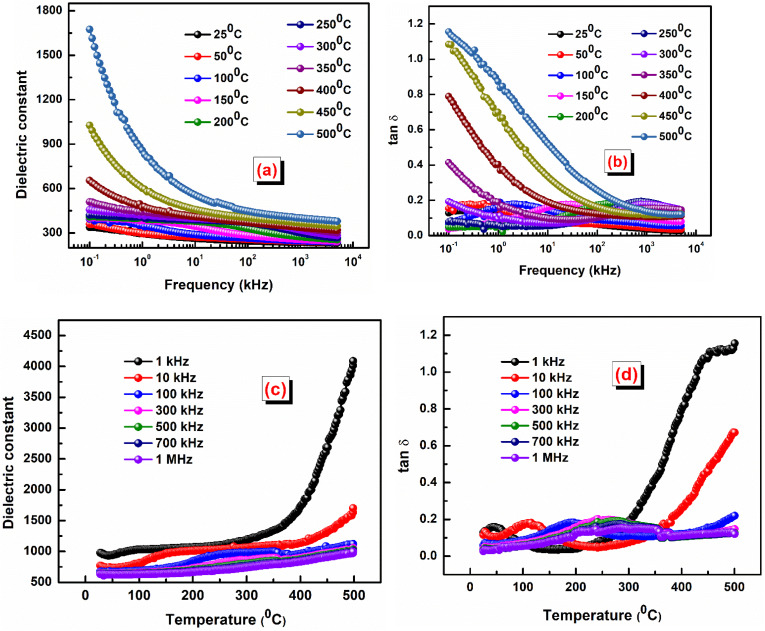
(a) Variation of the dielectric *versus* frequency and (b) tan *δ versus* frequency of the LBDO ceramic. (c) Variation of dielectric constant *versus* temperature and (d) tan *δ versus* temperature of the LBDO ceramic.

The variation of the dielectric constant with frequency is shown in Fig. 4(a) at some selected temperatures. The complex permittivity is expressed as ε*(*ω*) = *ε*′(*ω*) − j*ε*″(*ω*), where *ε*′ denotes the real part, and *ε*″ denotes the imaginary part of the permittivity.^28^ Furthermore, the real component can be determined using the relation *ε*′ = *Cdε*_0_*A*, where *ε*_0_ is the permittivity of free space. During the analysis of the dielectric constant with frequency, we will use the Debye equation: 
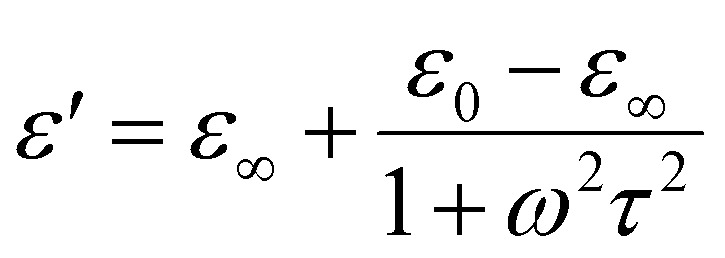
, where *τ* represents the relaxation time, *ε*_∞_ represents the dielectric constant at extremely high frequency, while *ε*_0_ represents the dielectric constant at extremely low frequency. Similarly, the expression for the dielectric loss can be calculated using the formula: tan *δ* = *ε*″/*ε*′.^29^

The real component of the dielectric response represents the material's ability to store energy, whereas the imaginary component corresponds to energy loss. The overall dielectric constant arises from the combined effects of electronic, ionic, dipolar, and space charge polarizations, each dominating over specific frequency ranges. At low frequencies, Maxwell–Wagner-type dispersion arises mainly due to grain boundary effects, leading to a high dielectric constant as all polarization mechanisms contribute.^30–32^ At higher frequencies, dipoles cannot reorient quickly enough with the alternating field, causing the dielectric constant to drop and become frequency-independent. Additionally, increased dipole energy at high frequencies reduces space charge polarization, further lowering the dielectric constant.^33^

The variation of the dielectric loss with frequency is shown in Fig. 4(b). Dielectric loss in a material arises from conduction, relaxation, and resonance mechanisms. Conduction loss, influenced by point defects like oxygen vacancies and impurities at phase boundaries, helps determine the material's electrical conductivity. Relaxation loss occurs when polarization cannot keep pace with a rapidly changing electric field, commonly seen at high frequencies. Resonance loss, on the other hand, appears at very high frequencies (∼10^13^ Hz or more) due to the vibrations of electrons, atoms, and molecules.^34^ At low frequencies, grain boundaries hinder charge carrier movement, increasing resistance and thus dielectric loss. Conversely, at high frequencies, grains offer lower resistance, resulting in reduced dielectric loss.^35^

Fig. 4(c) shows the variation of dielectric constant with temperature, while Fig. 4(b) depicts the temperature dependence of dielectric loss at some selected frequencies. The dielectric loss in materials originates from dc conduction, dipole relaxation, and space charge migration, representing the energy dissipated when polarization lags behind the applied electric field, mainly influenced by grain boundary effects. At low frequencies, the relatively higher dielectric loss is associated with the greater resistivity of grain boundaries compared to grains.^36^ The loss remains low at lower temperatures but increases with rising temperature, likely due to a mismatch between dipole relaxation and the frequency of the applied field.^37^ In the present study, the synthesized sample demonstrates a high dielectric constant along with low dielectric loss, highlighting its potential for energy storage applications.

### 3.4 AC conductivity study

**Fig. 5:**
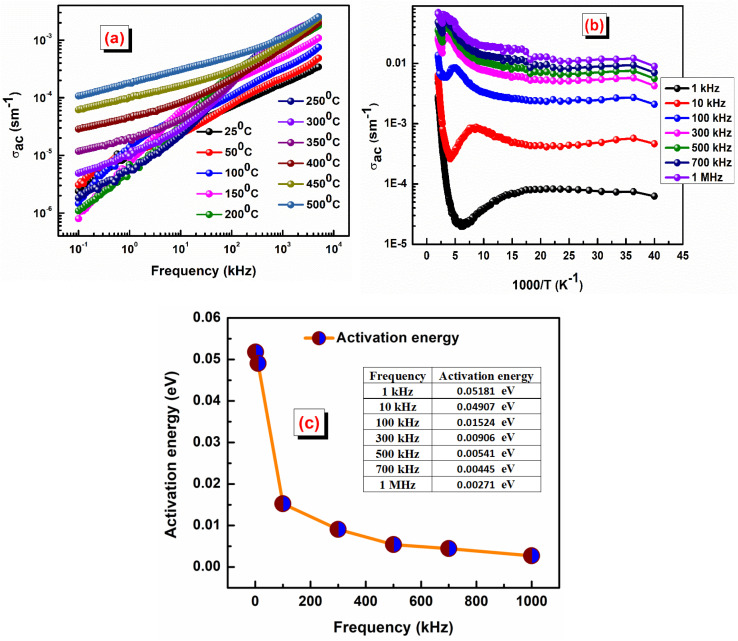
(a) Variation of ac conductivity *versus* frequency, (b) ac conductivity *versus* 1000/*T*, and (c) activation energy *versus* frequency of the LBDO ceramic.

The study of ac conductivity in ceramic materials reveals charge transport mechanisms and frequency-dependent behavior. It helps identify contributions from grain and grain boundary effects, hopping conduction, and polarization processes. Analyzing ac conductivity provides insight into electrical properties, crucial for optimizing ceramics in electronic and energy storage applications. Fig. 5(a) represents ac conductivity *versus* frequency, while Fig. 5(b) shows the variation of ac conductivity *versus* 1000/*T* at some selected frequencies. The ac conductivity can be calculated using the formula: *σ*_ac_(*ω*) = *ωε*_0_*ε*_r_ tan *δ*, all symbols have their usual meaning.^38^

The graph shows that ac conductivity increases with frequency, mainly due to ion collisions, cation disorder, and space charge effects. At higher temperatures, more electrons gain enough energy to move into the conduction band, raising conductivity. While ac conductivity increases linearly with temperature at low temperatures, it levels off at higher temperatures. The overlapping conductivity curves indicate space charge release and recombination at elevated temperatures.^39^Fig. 5(c) represents activation energy *versus* frequency of the LBDO ceramic. The activation energy was calculated by using the formula: 
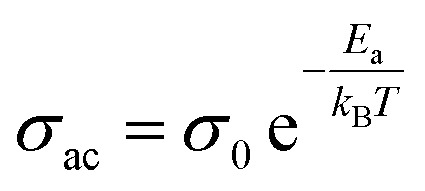
, where *E*_a_ = activation energy and all other symbols have their usual meaning. The plot of activation energy *versus* frequency reveals a decreasing trend of activation energy with the rise of frequency, supporting the presence of a thermally activated relaxation mechanism.^40^

### 3.5 Impedance study

**Fig. 6:**
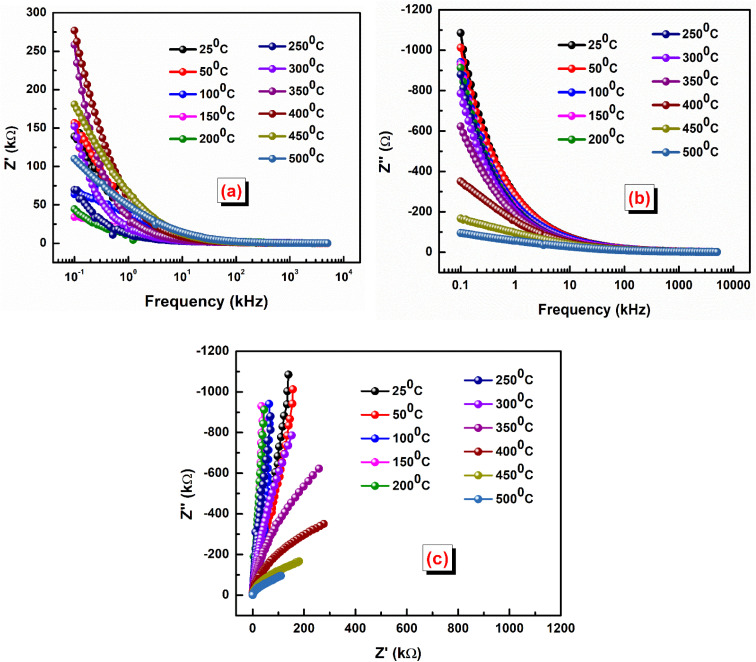
(a) Variation of *Z*′ *versus* frequency, (b) *Z*″ *versus* frequency, and (c) Nyquist plots of the LBDO ceramic.

An impedance study is crucial for ceramic materials as it reveals electrical properties, distinguishes grain and grain boundary effects, and helps understand conduction mechanisms, essential for designing advanced electronic and energy devices. The complex impedance Z* can be written as Z* = *Z*′ − j*Z*″, where *Z*′ represents real (resistance) and *Z*″ represents the imaginary part representing reactance.^41^Fig. 6(a) shows the real part of impedance (*Z*′) *versus* frequency, while Fig. 6(b) represents *Z*″ *versus* frequency at some selected temperatures. At low frequencies, the real part of impedance (*Z*′) decreases as temperature increases. This observation indicates an increase in the conduction process with rising temperature. This behavior aligns with the concept of negative temperature coefficient of resistance (NTCR), where the resistance of the material decreases as temperature increases.^42,43^ In this case, the decrease in *Z*′ suggests enhanced conductivity with temperature, which is characteristic of many semiconducting or conductive materials. As frequency increases, all the curves representing *Z*′ merge, irrespective of temperature. This phenomenon occurs due to the release of space charge. At higher frequencies, charge carriers experience less resistance to movement, and any charge accumulations that were present at lower frequencies are dissipated more rapidly.^44^ As a result, the impedance becomes less dependent on frequency and tends to converge to a similar value across different temperatures. A similar type of trend is observed from the analysis of imaginary plots of impedance.

The description of Fig. 6(c) indicates the presence of Nyquist plots, which are used to analyze impedance data in the complex plane. The presence of semicircular arcs in the Nyquist plots suggests a characteristic behavior associated with semiconducting materials.^45^ The description notes that the radius of the semicircular arcs decreases with increasing temperature. This observation supports the semiconducting nature of the material. As a result, the relaxation processes or charge transport mechanisms represented by the semicircular arcs become more efficient, leading to a reduction in the radius of the arcs as temperature increases.

### 3.6 Modulus study

**Fig. 7:**
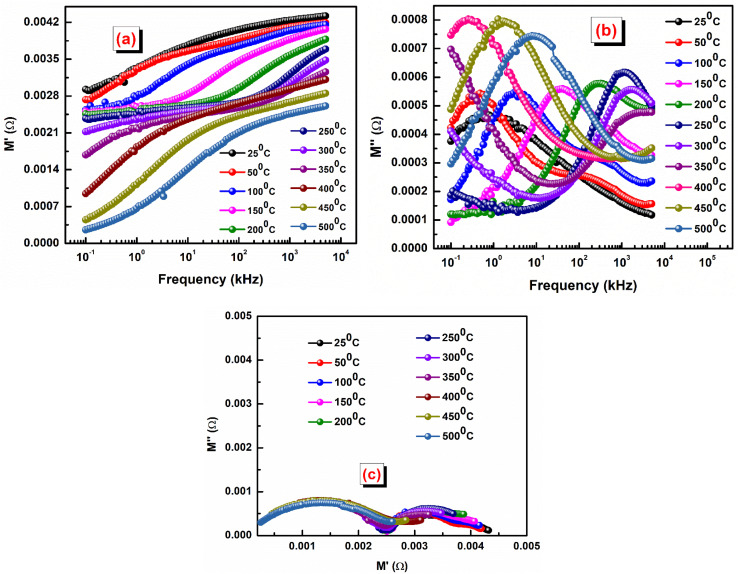
(a) Variation of *M*′ *versus* frequency, (b) *M*″ *versus* frequency, and (c) Cole–Cole plots of the LBDO ceramic.

The complex modulus can be calculated using the relation: M* = *M*′ + j*M*″ = 1/ε*; *M*′ = *ωC*_0_*Z*″ and *M*″ = *ωC*_0_*Z*′; 
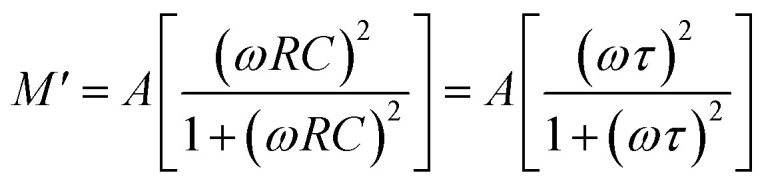
 and 
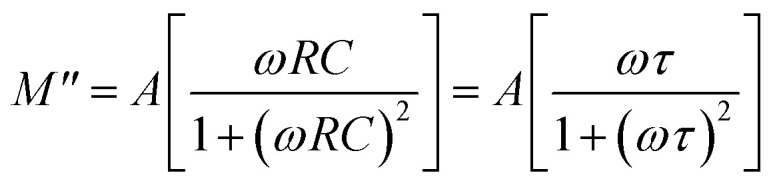
, where all symbols have their usual meaning.^46^Fig. 7(a) shows the variation of *M*′ *versus* frequency at some selected temperatures. In the low-frequency domain, the value of *M*′ approaches zero. This is likely due to the negligible electrode polarization effect. Electrode polarization occurs when charges accumulate at the electrode–electrolyte interface, creating a potential difference that opposes the applied electric field. At low frequencies, there's insufficient time for significant charge accumulation, resulting in a minimal electrode polarization effect. As frequency increases, the value of *M*′ rises. This could be attributed to various factors, but one potential explanation is the absence of a restoring effect from an induced electric field on the mobility of charge carriers. In other words, at higher frequencies, charge carriers experience less resistance to movement, leading to an increase in the real part of the modulus.^47^

In Fig. 7(b), the frequency dependence charts of the imaginary part of the modulus (*M*″) are presented at various temperatures ranging from 25 °C to 500 °C. Similar to *M*′, *M*″ also rises with increasing frequency. This behavior is often associated with the response of materials to an applied electric field at different frequencies. As the frequency increases, the response of the material becomes more pronounced, leading to higher values of *M*″. At a certain frequency, denoted as 
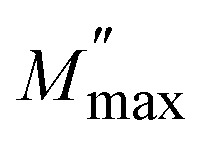
, the imaginary part of the modulus reaches its maximum value. This frequency likely corresponds to a specific characteristic of the material or the system under study. As the temperature increases, the peaks in *M*″ shift towards lower frequencies. This suggests that temperature affects the dynamics of the charge carriers or the material properties, altering the frequency at which the maximum response occurs.^48^ The behavior observed below the peak maximum frequency indicates long-range hopping of charge carriers. As the frequency increases beyond the peak maximum, the mobility of charge carriers becomes limited to potential wells, indicating short-range mobility. This transition from long-range to short-range mobility with increasing frequency provides insights into the charge transport mechanisms within the material. The shift of the peak towards higher frequencies with increasing temperature suggests that thermally stimulated processes, likely involving hopping mechanisms of charge carriers, dominate at higher temperatures. This indicates a temperature-dependent relaxation behavior within the material. The asymmetric broadening of the peak and the temperature dependence of its characteristics suggest a non-Debye relaxation behavior of the material. Peaks in *M*″ represent the actual dielectric relaxation process occurring within the material. The spread of the peak provides information about the distribution of relaxation time constants, indicating the grain and grain boundaries effect within the material.

The Cole–Cole plots shown in Fig. 7(c) are essential for understanding the electrical properties of the sample under study, particularly in terms of its semiconducting behavior. A Cole–Cole plot is a graphical representation commonly used in the analysis of dielectric materials. It typically displays the real part of the electrical modulus (*M*′) on the *x*-axis and the imaginary part (*M*″) on the *y*-axis. The plot often exhibits a semicircular arc shape. The presence of semicircular arcs in the Cole–Cole plots indicates a characteristic behavior associated with semiconducting materials.^49,50^ By analysing the shape and characteristics of the semicircular arcs in the Cole–Cole plots, researchers can extract valuable information about the electrical properties of the material. This includes parameters such as the relaxation time constant, distribution of relaxation times, and the nature of charge carrier dynamics. The presence of double peaks may indicate the existence of multiple relaxation processes within the material. Different relaxation mechanisms, such as dipolar relaxation, interfacial polarization, and electrode effects, can contribute to distinct relaxation timescales, leading to multiple peaks in the Cole–Cole plot. Finally, at low temperatures, heterogeneity (grain + grain boundary effects) is more pronounced, which produces “S-shaped” or multiple arcs, while at high temperatures, these differences become less significant (conductivities of grains and boundaries become similar) and the response merges into a single semicircular arc.

### 3.7 Resistance-dependent temperature

**Fig. 8:**
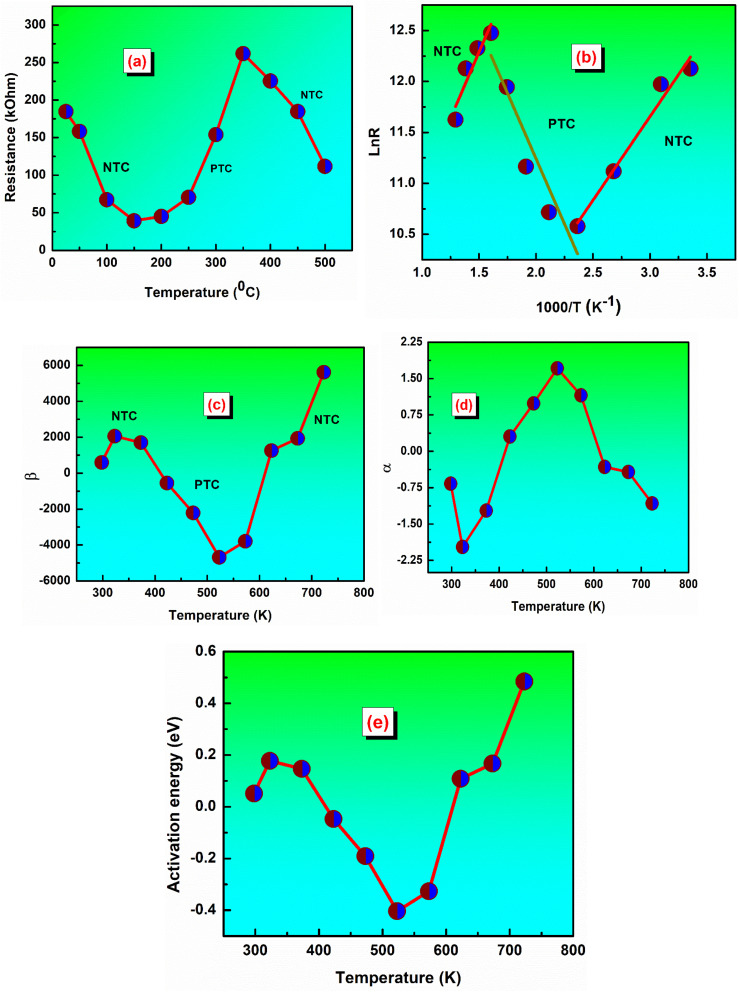
(a) Represents the value of resistance *versus* temperature, and (b) ln *R versus* 1000/*T* of the LBDO ceramic. (c) Shows the thermistor constant *versus* temperature, and (d) the sensitivity factor *versus* temperature of the polycrystalline LBDO ceramic. (e) Activation energy *versus* temperature of the LBDO ceramic.

Many ceramics, like thermistors, show strong resistance changes with temperature, making them useful for temperature sensing or control.^51^ The resistance values were calculated from the Nyquist plots at different temperatures. Fig. 8 shows the variation of the resistance *versus* temperature. Thermistive materials fall into two primary categories: NTCR, where resistance decreases with rising temperature, and PTCR, where the resistance increases with rising temperature.^52^

It is observed that resistance decreases from the temperature of 25 °C to 150 °C, following the NTC thermistor character.^53^ Again, the resistance increases from 200 °C to 350 °C, following the PTC thermistor character. Interestingly, a fall in the value of resistance is observed with the increase in the temperature from 350 °C to 500 °C, thereby confirming the NTCR nature. Therefore, the studied material has two NTC thermistor ranges, which may be treated as one suitable candidate for a temperature sensor device in both low and high temperature ranges. Fig. 8(b) shows a plot between ln *R versus* 1000/*T* of the LBDO ceramic. From the analysis, it is found that the plot has two distinct regions and confirms both NTC and PTC nature in the said range of temperature.^54^

The thermistor constant (*β*) is an important parameter that characterizes the relationship between temperature and resistance in NTC ceramic thermistors. The thermistor constant can be calculated using the formula: 
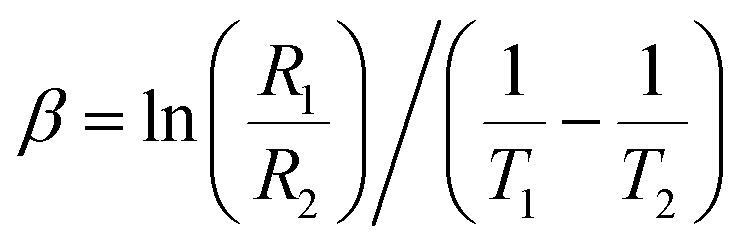
, where *R*_1_ and *R*_2_ denote the resistances measured at the corresponding temperatures *T*_1_ and *T*_2_.^55^ In this study, the thermistor constant is determined in both the NTC and PTC regions, as illustrated in Fig. 8(c). According to the literature, the thermistor constant typically ranges from 15 000 to 18 000 K for high-temperature thermistor applications, and from 1500 to 5000 K for low-temperature thermistor applications.^56,57^ Interestingly, the thermistor constant of the investigated sample ranges from 593 K to 5620 K, indicating that the prepared sample is appropriate for low-temperature thermistor applications.^58^

The sensitivity factor is mathematically expressed as the rate of change of the logarithm of resistance with respect to temperature. It indicates how efficiently a thermistor senses and reacts to temperature variations, making it an essential parameter for its design and performance. For an NTC thermistor based on the beta model, the sensitivity factor is given by: *α* = (−*β*/*T*^2^) × 100, where all symbols retain their usual meanings.^59^Fig. 8(d) illustrates the variation of the sensitivity factor with temperature. Higher sensitivity values indicate improved resolution in detecting minor temperature variations.^60^ In this study, the sample exhibits maximum sensitivity at 523 K, suggesting that it can serve as an effective temperature sensor at this temperature. Activation energy is the minimum energy required to initiate the movement of charge carriers that allows electrical conduction in a ceramic material.

Fig. 8(e) shows the plot between activation energy *versus* temperature in LBDO ceramic. Activation energy refers to the minimum energy necessary to enable the movement of charge carriers responsible for electrical conduction in ceramic materials. It can be determined using the relation *E*_a_ = *k*_B_ × *β*, where *k*_B_ is the Boltzmann constant (1.38 × 10^−23^ J K^−1^).^61^ In the present study, the activation energy of NTC ceramic thermistors typically lies in the range of 0.051 to 0.484 eV. All the thermistor parameters like temperature, resistance, thermistor constant, sensitivity factor, and activation energy are calculated using the above-mentioned formula and listed in Table 1. Therefore, based on the above analysis of thermistor parameters and their variation, it can be concluded that the prepared sample is well-suited for NTC thermistor applications.

**Table 1:** Lists the thermistor parameters of the LBDO ceramic

Temp. (°C)	Temp. (K)	1/*T*	1000/*T*	*R* (Ω)	ln *R*	*β*	*α*	*E*_a_
25	298	0.00336	3.3557	184 649.1	12.12621	592.3	−0.667	0.05109
50	323	0.0031	3.09598	158 320.3	11.97238	2059.4	−1.973	0.17762
100	373	0.00268	2.68097	67 351.6	11.11768	1700.7	−1.222	0.14669
150	423	0.00236	2.36407	39 290.2	10.57873	−549.8	0.307	−0.04742
200	473	0.00211	2.11416	45 077.1	10.71613	−2214.4	0.989	−0.19099
250	523	0.00191	1.91205	70 524.3	11.16371	−4682.5	1.711	−0.40387
300	573	0.00175	1.7452	154 041.3	11.94498	−3786.6	1.153	−0.32659
350	623	0.00161	1.60514	261 802.9	12.47535	1255.5	−0.323	0.10829
400	673	0.00149	1.48588	225 396.1	12.32561	1935.1	−0.427	0.1669
450	723	0.00138	1.38313	184 753.3	12.12678	5619.1	−1.074	0.48465
500	773	0.00129	1.29366	111 755.1	11.62407	—	—	—

## 4. Conclusion

The orthorhombic LBDO ceramic was synthesized *via* the conventional solid-state reaction method. W–H plot is drawn to find average crystallite size of 49.1 nm, lattice strain of 0.000876, and dislocation density of 4.14 × 10^14^ m^−2^ in the reported sample. SEM analysis revealed uniformly distributed grains with well-defined grain boundaries, which facilitate the conduction mechanism. The EDX spectrum confirmed the presence of all constituent elements (La, Bi, Dy, and O) in both weight and atomic percentages. Analysis of the dielectric constant variation with frequency indicated Maxwell–Wagner type dielectric dispersion, while impedance–frequency studies demonstrated that the sample exhibits NTCR behavior. Electrical modulus analysis confirms the presence of a non-Debye type relaxation process. The variation of ac conductivity with frequency and temperature indicates that the sample follows a thermally activated conduction mechanism. The reduction in grain boundary resistance, evidenced by the shrinking semicircle radius in Nyquist plots with rising temperature, further supports its semiconducting behavior, making it a potential candidate for energy storage applications. Additionally, Cole–Cole plot analysis highlights the contributions of both grains and grain boundaries to the conduction process and reinforces the semiconducting nature inferred from the Nyquist analysis. Future studies should focus on compositional optimization through controlled substitution at B-sites to tailor the electrical and dielectric properties. Extended temperature- and frequency-dependent studies, including long-term thermal cycling and aging tests, are necessary to evaluate device stability. Despite these encouraging results, the present study has certain limitations. The investigation was restricted to a single composition, and hence the influence of compositional variation on structural, dielectric, and electrical properties remains unexplored. Again, the absence of direct energy storage parameters such as polarization–electric field (*P*–*E*) hysteresis loops also limit a comprehensive assessment of energy storage performance. However, the present study, the resistance *versus* temperature relationship confirms an NTC thermistor application in low range of temperature, particularly from 25 °C to 150 °C, highlighting its potential use in temperature sensor devices.

## Conflicts of interest

The authors report no conflicts of interest related to this work.

## Data Availability

Data will made available on reasonable request from the authors.
